# Cerebrospinal Fluid Concentrations of Bedaquiline, Pretomanid, and Linezolid During Curative Treatment of Fluoroquinolone-Resistant Pre–Extensively Drug-Resistant Tuberculous Meningitis

**DOI:** 10.1093/ofid/ofag445

**Published:** 2026-07-29

**Authors:** Adrian Robert Tramontana, Andrew Burke, Maria Globan, Brett McWhinney, Aaron E Bloch, Benjamin J Smith, Justin T Denholm, Connor James Wright, James S Molton

**Affiliations:** Department of Infectious Diseases, Western Health, Footscray, Victoria, Australia; Department of Infectious Diseases, The Prince Charles Hospital, Chermside, Queensland, Australia; Victorian Infectious Diseases Reference Laboratory, Melbourne, The Peter Doherty Institute for Infection and Immunity, Melbourne, Victoria, Australia; Analytical Chemical Unit, Central Laboratory, Health Support Queensland, Pathology Queensland, Herston, Queensland, Australia; Department of Infectious Diseases, Western Health, Footscray, Victoria, Australia; Department of Infectious Diseases, Western Health, Footscray, Victoria, Australia; The Victorian Tuberculosis Program, Melbourne Health, Melbourne, Australia; Department of Infectious Diseases, Western Health, Footscray, Victoria, Australia; Department of Infectious Diseases, Western Health, Footscray, Victoria, Australia

**Keywords:** bedaquiline, cerebrospinal fluid, multi-drug resistant tuberculosis/drug therapy, pretomanid, tuberculous meningitis

## Abstract

**Background:**

Bedaquiline, pretomanid, and linezolid (BPaL) is a 6-month regimen that has revolutionized treatment of multidrug-resistant (MDR) tuberculosis (TB). However, there is limited evidence of using BPaL to treat MDR tuberculous meningitis (TBM), a devastating illness that has high risk of mortality and permanent disability.

**Methods:**

A patient with fluoroquinolone-resistant pre–extensively drug-resistant (pre-XDR) TBM was cured with a 10-month regimen based on BPaL with clofazimine and cycloserine. Total concentrations of bedaquiline, pretomanid, and linezolid were measured in plasma and cerebrospinal fluid (CSF) via ultraperformance liquid chromatography. Unbound concentrations were estimated from plasma concentrations and CSF concentrations normalized to plasma protein concentrations using the Winter–Tozer formula. AUC_24_ (24-hour area under the curve) was calculated using the trapezoidal method.

**Results:**

The CSF:plasma unbound AUC_24_ was 1.48 and 0.99 for pretomanid and linezolid, respectively. Pharmacokinetic/pharmacodynamic targets were achieved for pretomanid with CSF concentration above the critical concentration of 0.5 mg/L throughout most of the dosing interval and linezolid AUC_24_/minimum inhibitory concentration (MIC) of 171 for an MIC of 0.5 mg/L. However, CSF concentrations of pretomanid and linezolid may be below target for strains with MICs >0.5 mg/L. Estimated unbound peak concentrations in CSF in week 21 were comparable to plasma (0.0034 mg/L and 0.0032 mg/L, respectively), suggesting that therapeutic central nervous system concentrations of bedaquiline were achieved.

**Conclusions:**

Fluoroquinolone-resistant pre-XDR TBM was cured with 10 months of treatment based on BPaL with clofazimine and cycloserine. BPaL achieved therapeutic concentrations in CSF for most TB strains. Further research is required into optimal treatment and dosing of drug-resistant TBM.

## Teaser

Fluoroquinolone-resistant pre-XDR tuberculous meningitis was cured with a 10-month regimen based on bedaquiline, pretomanid, linezolid, and clofazimine with cycloserine. Central nervous system concentrations of bedaquiline, pretomanid, and linezolid were therapeutic except for TB strains with pretomanid or linezolid MIC >0.5 mg/L.

The current recommended treatment of multidrug-resistant (MDR) tuberculosis (TB) is a 24 week all-oral regimen consisting of bedaquiline, pretomanid, and linezolid (BPaL) with or without moxifloxacin [1, 2]. Favorable outcomes have been reported in >90% of patients with MDR pulmonary TB. However evidence for BPaL in central nervous system (CNS), osteoarticular, and disseminated TB is limited and not recommended by guidelines [3–6]. BPaL appears equally effective in TB osteomyelitis with successful outcomes for all 5 patients treated in a US case series [3]. For CNS TB, there is one reported case of sterile cerebral tuberculoma in a patient previously treated with BPaL during the Nix-TB trial [7].

Tuberculous meningitis (TBM) is the most severe form of TB, with 27%–40% mortality in treated adults and 32% physical disability in survivors [8–10]. Drug resistance compounds poor outcomes with a further 7-fold increase in mortality [11]. Contributing to poor outcomes of drug-resistant TBM is lack of data and guidance on effective antimicrobial therapy [1, 2]. Linezolid has shown value in an observational study of TBM and continues to be studied as adjunctive treatment in trials [12–14]. An early report of bedaquiline concentrations in cerebrospinal fluid (CSF) suggested poor penetration into the CNS [15]. More recent evidence suggests that bedaquiline and pretomanid achieve therapeutic CNS concentrations [16–18]. Data on use of BPaL in TBM are predominately from animal models, and further clinical and pharmacokinetic data in humans with TBM are needed.

We report here a cure of fluoroquinolone-resistant pre–extensively drug-resistant (pre-XDR) TBM in a 35-year-old woman using a 10-month regimen of bedaquiline, pretomanid, linezolid, and clofazimine (BPaLC) plus cycloserine, and we provide data on CSF concentrations of pretomanid, bedaquiline, and linezolid. Her son's concurrent treatment of congenital TB has been reported separately [19].

## Clinical Details

**Figure 1:**
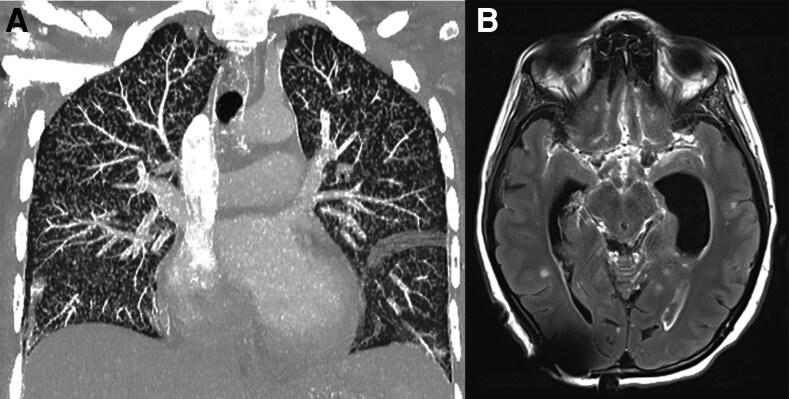
Computed tomography (CT) of the chest and magnetic resonance imaging (MRI) of the brain. *A*, Coronal chest CT scan demonstrating diffuse miliary pulmonary infiltrate. *B*, Axial fluid-attenuated inversion recovery MRI of the brain with contrast demonstrating multiple enhancing nodules in the leptomeninges of the basal cisterns and subcortical white matter, hydrocephalus of the temporal horn of the left lateral ventricle, and artefact in the parietal lobe from a ventriculoperitoneal (VP) shunt in the right lateral ventricle. The VP shunt into the left lateral ventricle was inserted later.

In January 2022 a 35-year-old woman without human immunodeficiency virus presented with fevers, cough, headache, vaginal discharge with abdominal pain, and tenderness over the uterus 9 days after premature delivery of a boy conceived through in vitro fertilization. Chest computed tomography demonstrated miliary nodules throughout both lungs (Figure 1), and histological sections of retained products of conception demonstrated necrotizing granulomas. Initially she had no neurological symptoms and was commenced empirically on first-line TB therapy. TB cultures and molecular testing via Xpert MTB/RIF Ultra of sputum and bronchoalveolar lavage fluid were negative. One week later, her Glasgow Coma Scale score decreased to 11. Imaging demonstrated obstructive hydrocephalus and acute subcortical infarcts associated with vasculitis from basal meningitis (Figure 1). Further molecular testing via Xpert MTB/RIF Ultra and Xpert MTB/XDR (Cepheid, Sunnyvale, CA, USA) assays on retained products of conception detected *Mycobacterium tuberculosis* and identified rifampicin, isoniazid, and fluoroquinolone resistance mutations. She was commenced on an MDR-TB regimen following collection of CSF, blood, urine, and repeat endometrial and sputum samples. TB was cultured from all these specimens, and Xpert MTB/RIF Ultra detected TB in the CSF, sputum, and endometrial tissue samples. Microscopy for acid-fast bacilli was negative on all specimens. CSF cultures for TB converted to negative on day 11 of active antibiotic therapy.

**Figure 2.**
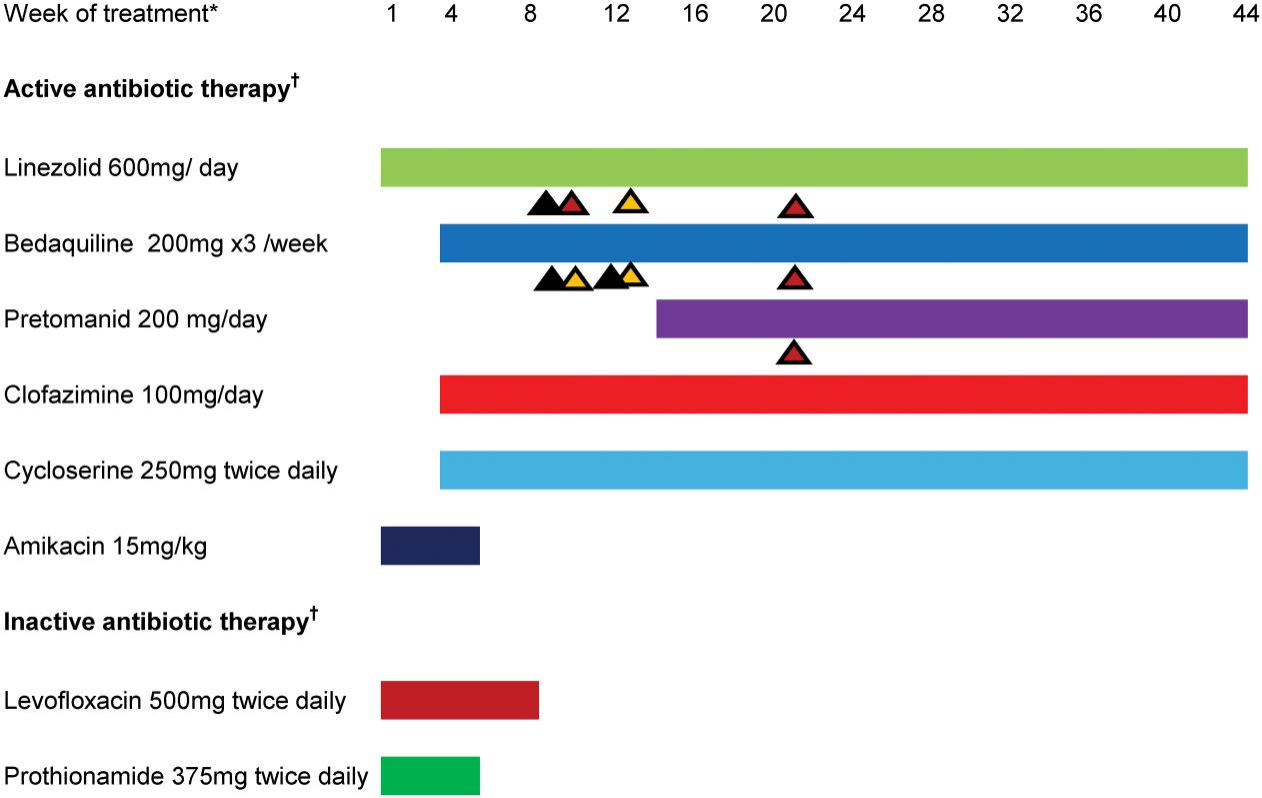
Timeline of antimicrobial therapy and measurement of drug concentrations. *Start of treatment taken as first day of an active antibiotic. Isoniazid, rifampicin, ethambutol, and pyrazinamide administered prior to identification of rifampicin resistance have not been included. ^†^Active antimicrobial therapy is antibiotic therapy without in vitro phenotypic and genotypic resistance. Bedaquiline dose was 400 mg/day for the first 2 weeks of therapy. Weight at start of antimicrobial therapy was 49 kg. Serial plasma and cerebrospinal fluid (CSF) samples were collected at time 0, 2, and 6 hours in week 21. 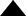 plasma, 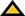 CSF, and 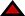 plasma and CSF samples collected.

Antimicrobial therapy following identification of genotypic rifampicin resistance is outlined in Figure 2. Initially, linezolid and amikacin were the only active antibiotics administered until bedaquiline and clofazimine were added on day 22; amikacin was ceased after 34 days and replaced with cycloserine. Pretomanid was sourced and commenced 13 weeks into active antimycobacterial therapy. Levofloxacin and prothionamide were administered until high-level fluoroquinolone and ethionamide resistance was demonstrated by phenotypic testing. Antibiotics were ceased after 29 weeks of pretomanid and 43 weeks total treatment. Side effects from treatment included mild elevation of liver transaminases and abdominal pain.

Ancillary management included bilateral ventriculoperitoneal shunts and immunomodulation with prednisolone and thalidomide. Associated with completing an initial 12-week tapered course of prednisolone was a decline in neurological function and increase in CSF white cell count (Table 1). Prednisolone was recommenced at 20 mg per day and thalidomide was then added after 18 weeks of antibiotics to facilitate tapering of prednisolone dose. Following a rebound in CSF white cell count, thalidomide dose was increased to 300 mg/day and then prednisolone dose was tapered to 5 mg/day and continued till the end of antibiotic therapy. Thalidomide was ceased 1 month after completing antibiotics.

**Table 1.** Cerebrospinal Fluid Microscopy, Culture, and Biochemistry

Investigation	Time From Start of Active Treatment					
	Day 2	Week 2	Week 9	Week 12	Week 17	Week 21
Site	Lumbar	Ventricle	Lumbar	Lumbar	Lumbar	Lumbar
Glucose, mmol/L	3.0	3.3	1.8	1.6	1.2	1.4
Protein, g/L	7.02	0.41	6.76	6.95	7.48	6.90
RBCs, 10^6^/L	9	1000	ND	510	590	27
WBCs, 10^6^/L	82	7	ND	680	65	790
Neutrophils	41	0	…	20	0	198
Mononuclear	41	7	…	660	65	592
AFB smear	−	−	−	−	−	−
AFB culture	+	−	−	−	−	−
Prednisolone, mg/d^a^	50	50	20	0	20	15

Abbreviations: AFB, acid-fast bacilli; ND, not done; RBCs, red blood cells; WBCs, white blood cells.

^a^Prednisolone dose administered on day of cerebrospinal fluid sampling.

At the end of treatment, she had severe neurological disability attributable to TBM and its complications. On last follow-up 40 months after treatment, she had no signs of recurrent TB. Modest improvement in neurological function continues with a recent milestone of walking 20 steps with assistance. However, she requires ongoing care for activities of daily living and a motorized wheelchair for mobility.

## MATERIALS AND METHODS

### Microbiology

Initial molecular testing was performed directly on paraffin-embedded endometrial tissue via the Xpert MTB/RIF Ultra and MTB/XDR assays that had been validated for test performance on sample types other than those recommended by the manufacturer. Subsequent molecular testing was performed on fresh samples of CSF, blood, urine, fresh tissue, and sputum using GeneXpert. These samples were also cultured to obtain an isolate for phenotypic drug susceptibility testing (DST) and whole genome sequencing analysis. Phenotypic DST was performed via the Bactec MGIT 960 system (Becton Dickinson).

### Drug Concentrations and Pharmacokinetics

Plasma samples were collected into tubes containing ethylenediaminetetraacetic acid at times shown in Figure 2. Single CSF samples were collected via lumbar puncture, and serial samples for pretomanid, bedaquiline, and linezolid concentrations were collected via a temporary lumbar catheter during week 21 of treatment. All samples were centrifuged and frozen at −70°C prior to being transported. Drug concentrations were measured using an Acquity ultra performance liquid chromatography (UPLC) system, an Acquity UPLC high strength silica (HSS) T3 column (1.8 µM, 2.1 × 100 mm), MassLynx V4.2 software, and a Xevo TQD mass spectrometer (Waters, Milford, MA, USA). Plasma (50 µL) was mixed with 200 µL of methanol, containing deuterated internal standards for each respective drug, and centrifuged for 10 minutes. The supernatant (1 µL) was injected onto the system and chromatographically resolved using a Waters 2.1 × 50-mm HSS 1.8-µm column. Each drug was linear over the analytical range up to 50 mg/L. The coefficient of variation (%CV) ranged from 1.1% to 6.5% and from 1.5% to 7.4% for intra- and interrun, respectively, across 3 concentrations. Recovery for specific analytes ranged between 97% and 103%. No interferences were found when extracting plasma samples containing various other drugs encountered within our laboratory. All drugs were stable postextraction for a minimum of 24 hours.

Area under the curve (AUC) values were calculated using the trapezoidal method. When there was more than one concentration at a time point, AUC was calculated using the average of concentrations at that time point. To avoid overestimating linezolid AUC in CSF, we assumed that marginally higher CSF than plasma concentrations were maintained between measured concentrations at 7 and 24 hours postdose and estimated AUC in CSF using 12- and 16-hour plasma concentrations. To account for considerable differences in CSF and plasma protein concentrations for highly protein-bound pretomanid and bedaquiline, total CSF concentrations were normalized to plasma protein concentrations using the Winter–Tozer formula


$$C_{tn}=\frac{C_{to}}{\;f_{u}+f_{p}\frac{P_{csf}}{P_{\;pl}}}$$

where C_tn_ and C_to_ are normalized and observed total concentration, f_u_ and f_p_ are unbound and protein-bound fractions in plasma, and P_csf_ and P_pl_ are CSF and plasma protein concentrations, respectively [20]. Unbound fractions of pretomanid, bedaquiline, and linezolid were derived from previously reported protein binding of 15%, 0.1%, and 95% of total concentrations, respectively [17, 18, 21]. Estimated unbound concentrations derived from total plasma concentration and normalized CSF concentrations were used for comparison with pharmacokinetic/pharmacodynamic (PK/PD) targets of pretomanid (time greater than the minimum inhibitory concentration [MIC] >48%) and linezolid (24-hour AUC [AUC_24_]/MIC >125) [22, 23].

## RESULTS

Xpert MTB/RIF Ultra assay on paraffin-embedded endometrial tissue detected *Mycobacterium tuberculosis* DNA (low level) together with a mutation within the *rpoB* gene, with subsequent testing within the Xpert MTB/XDR assay detecting isoniazid and fluoroquinolone mutations.

Phenotypic resistance was demonstrated to high-level isoniazid, rifampicin, ethambutol, pyrazinamide, high-level fluoroquinolones, and ethionamide. Whole genome sequencing classified the isolate as an East Asian (lineage 2) strain with resistance mutations detected for rifampicin (*rpoB* p.Ser450Leu), isoniazid (*kat*G p.Ser315Thr), ethambutol (*emb*B p.Met360Val), fluoroquinolones (*gyr*A p.Asp94Gly), and para-aminosalicylic acid (*thy*X c.-16C > T, ThyA chromosome:g.3073680_3074470del), with concordance shown between phenotypic and genotypic results. Phenotypic DST demonstrated susceptibility to bedaquiline, pretomanid tested at 0.5 mg/L, and linezolid tested at 0.5 mg/L and the critical concentration of 1.0 mg/L [24, 25].

**Figure 3.**
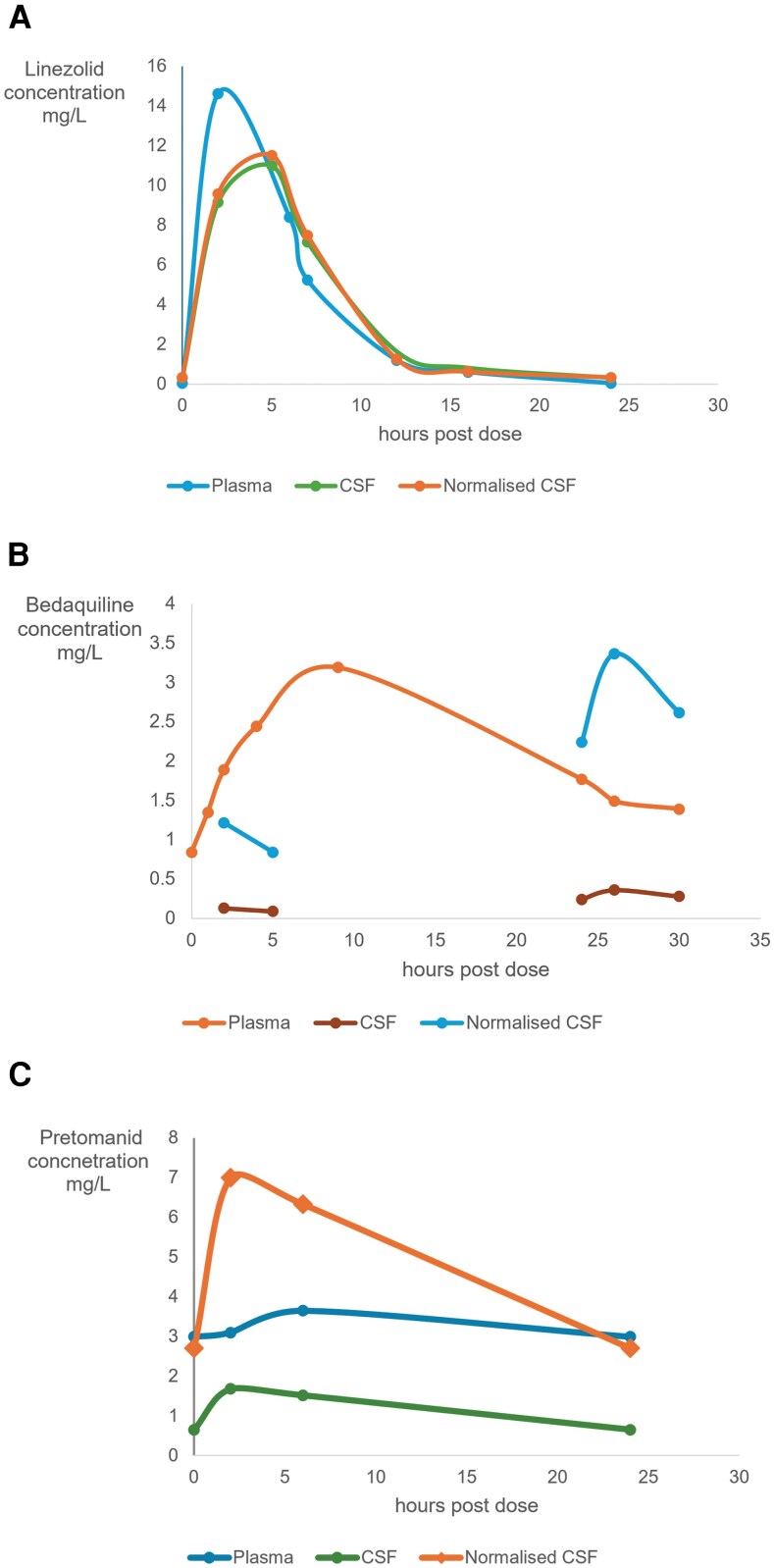
Linezolid, bedaquiline, and pretomanid concentrations in plasma and cerebrospinal fluid (CSF). *A*, Linezolid concentration. *B*, Bedaquiline concentration. *C*, Pretomanid concentration. Due to overlap with CSF concentrations, normalized CSF concentrations were not included for linezolid. Bedaquiline concentrations at zero to 5 hours postdose were from samples collected in weeks 9–12 of treatment and 24 to 20 hours postdose were from samples collected in week 21 of treatment.

Plasma and CSF drug concentrations for bedaquiline, linezolid, and pretomanid are shown in Figure 3 and Table 2. Bedaquiline CSF concentrations were higher in samples collected in week 21 of treatment compared to samples collected in weeks 9–12 of treatment (Figure 3*B*). CSF concentrations normalized to serum protein were calculated when CSF protein concentration was 6.9 g/L, total serum protein 65 g/L, and serum albumin 34 g/L.

**Table 2.** Plasma and Cerebrospinal Fluid Concentrations of Linezolid, Bedaquiline, and Pretomanid

Concentration	Linezolid	Bedaquiline	Pretomanid
Plasma peak concentration, mg/L	14.63	3.19	3.65
Unbound plasma peak concentration, mg/L	13.9	0.0032	0.55
Time to peak, h	2	9	6
CSF peak concentration, mg/L	11.0	0.36^a^	1.68
Normalized CSF peak concentration, mg/L^b^	11.5	3.36	6.99
Unbound CSF peak concentration, mg/L^c^	10.9	0.0034	1.05
Time to peak, h	5	26^a^	2
Plasma trough concentration, mg/L	0.04–0.10	0.84	2.99
CSF trough concentration, mg/L	0.32	NA	0.65
Normalized CSF trough concentration, mg/L	0.33	NA	2.71
Unbound CSF trough concentration, mg/L	0.31	NA	0.41
Plasma AUC_24_, mg/L.h	90	87^a^	79
Unbound plasma AUC_24_, mg/L.h	85.5	0.087	11.9
CSF AUC_24_, mg/L.h	89	NA	28
Normalized CSF AUC_24_, mg/L.h	90	NA	118
Unbound CSF AUC_24_, mg/L.h	85.5	NA	17.7
AUC_24_ CSF:plasma	0.99	NA	1.48

Abbreviations: AUC_24_, 24-hour area under the curve; CSF, cerebrospinal fluid; NA, not available.

^a^Highest measured concentration was 26 hours postdose in week 21 of treatment. Peak concentration may have been at earlier time points.

^b^CSF concentrations normalized to plasma protein concentrations.

^c^Unbound CSF concentrations were estimated from CSF concentrations normalized to plasma.

In both plasma and CSF, unbound pretomanid concentrations were estimated to be above the lower critical concentration of 0.5 mg/L throughout most of the dosing interval, whereas unbound bedaquiline concentrations were below the critical concentration throughout the dosing interval [25, 26]. Unbound linezolid AUC_24_/MIC was 85.5 and 171 for an MIC of 1.0 (critical concentration) and 0.5 mg/L, respectively [24].

## DISCUSSION

MDR TBM is a devastating illness that has high mortality and permanent disability [2, 8, 9, 11]. More data on the use of BPaL are required to determine if the significant improvements in successful treatment of MDR-TB pulmonary disease can be extended to those who have drug-resistant meningitis [2, 27]. Our report of CNS TB cured by a BPaL-based regimen complemented by data on CSF antibiotic concentrations provides support for the use of BPaL in TBM. In contrast to the previous report of an occult cerebral tuberculoma identified after TB treatment, our patient had extensive TBM and neurological complications before commencing effective TB therapy [7].

Prior to the addition of pretomanid and CNS accumulation of bedaquiline, linezolid was likely the core of our patient's early CNS treatment. Data on effectiveness of linezolid in TBM are limited. Addition of linezolid to an active regimen has shown to improve surrogate endpoints while improvement in survival was reported in one small series of rifampicin-resistant TBM [13, 14, 28]. Amikacin in the setting of inflammation has good CNS penetration and was potentially an important early companion agent [29, 30]. Cycloserine had uncertain value in our patient's treatment despite reportedly having good CNS penetration [29]. For our patient's dose of 250 mg twice daily, Deshpande et al reported 60% and >90% probability of target attainment for bactericidal activity for the critical concentration of 16 mg/L and MIC of 8 mg/L, respectively [31]. We were reluctant to increase the dose due to challenges of identifying toxicity in among poor neurological function. In hindsight, therapeutic drug monitoring for cycloserine would have been valuable. The value clofazimine had in our patient's CNS TB is also uncertain, with previous reports of low CSF concentrations vastly underestimating CNS penetration by not taking account of high protein binding and long plasma-to-CNS equilibration time [29, 32, 33].

Linezolid had excellent CNS penetration in our patient with a CSF:plasma ratio of 0.99 and AUC_24_/MIC above the target of 125. However, if the linezolid MIC was above the MIC_90_ of 0.5 mg/L, AUC_24_/MIC would have been below target in the CNS [22]. Other investigators have found substantial variability in linezolid plasma concentrations, CNS penetration, and hence CSF concentrations [13, 21, 29, 32, 34]. We therefore recommend therapeutic drug monitoring to achieve PK/PD targets in the CNS while limiting risk of linezolid's dose-related toxicity. Some of the reported variability in CNS pharmacokinetics may be due to protracted CNS penetration, and measuring linezolid CSF concentrations later in the dosing interval may avoid underestimating CSF AUC_24_ [18, 34].

After taking into account protein binding, we were able to demonstrate that normalized pretomanid concentrations were higher in CSF than plasma. For highly protein-bound drugs, normalized concentrations provide a conceptual and likely more accurate estimate of unbound drug concentrations relative to plasma in low-protein fluid such as CSF [20]. Our estimate of a normalized pretomanid CSF-to-plasma concentration ratio of 1.5 is similar to brain tissue-to-plasma ratios obtained in mice and healthy human volunteers using positron emission tomography–computed tomography, which supports the use of this ratio as a valid comparator [18].

Our estimated unbound pretomanid concentrations in CSF exceeded the PK/PD target of 48% time above MIC for the proposed critical concentration of 0.5 mg/mL [23, 25]. However, estimated unbound concentrations in both plasma and CSF did not reach the World Health Organization's upper critical concentration of 2.0 mg/mL. The probability of achieving target pretomanid CNS concentrations is favorable, with most TB strains having an MIC <0.5 mg/mL [25]. TB lineage 1 isolates are the exception, with most having an MIC of 1.0 mg/mL. Apparent equivalent clinical success with lineage 1 infections may be due to most of the clinical data relating to pulmonary disease where lung tissue concentrations are significantly higher than plasma and extrapulmonary tissues [18]. Caution should be exercised with pretomanid in extrapulmonary TB from lineage 1 strains.

In contrast to Akkerman and colleagues' report of an inability to detect bedaquiline in patients’ CSF [15], we were able to detect relatively high CSF concentrations. The impairment of our patient's blood-brain barrier may have significantly increased CNS penetration with CSF concentrations significantly higher than maximum concentrations reported in patients without meningitis [17]. Normalized CSF concentrations in the first 12 weeks of treatment were consistent with a brain-to-plasma ratio of 15%–35% seen in mice after a single dose of bedaquiline [16, 35]. Following CNS accumulation over 21 weeks, normalized CSF concentrations exceeded plasma concentrations, inferring that therapeutic CNS concentrations were potentially achieved. Unbound bedaquiline concentrations are usually only above MICs in lysosomes of macrophages, and further data are required to determine if normalized CSF concentrations can provide a valid proxy for concentrations in microglial lysosomes similar to correlation of plasma AUC/MIC targets for effectiveness in pulmonary TB [26, 36].

It is important to highlight treatment duration. Our patient was cured of TBM within 10 months of treatment and 6 months of pretomanid. This is significantly shorter, less toxic, and overall more favorable than the 12- to 24-month regimen recommended for MDR TBM [2, 37]. It is even shorter than the 12-month regimen usually given for drug-susceptible TBM. However, TBM is usually a paucibacillary disease and 6 months of first-line TB therapy is likely sufficient for most drug-susceptible cases [37]. Although BPaL had reduced early bactericidal activity compared to first-line TB therapy in a murine TBM model, it has superior sterilizing activity, which is the main determinant of total treatment duration [18]. Additionally, the long terminal half-life of bedaquiline provides ongoing activity months after completing treatment [38].

There are a number of limitations to our findings. First, only total drug concentrations in plasma and CSF from a single patient were measured. The role of cycloserine and short course of amikacin in achieving cure of this case is uncertain. Additionally, the relatively short duration of therapy for TBM in our case may not be applicable to other cases of CNS TB, such as large cerebral abscesses with higher bacillary burdens or CNS infections with lineage 1 strains with pretomanid MICs of 1.0–2.0 mg/mL. Individuals with TBM may also exhibit varying degrees of meningeal inflammation, which may impact CNS drug penetration, limiting the applicability of drug levels recorded from a single case. However, we provide the second published report of clinical success of BPaL for CNS TB and add to the limited human data on CNS concentrations of bedaquiline and pretomanid in patients with TBM. Normalized CSF concentrations may have imprecisions due to differences in protein binding between CSF and plasma.

In conclusion, our patient with fluoroquinolone-resistant pre-XDR TBM was cured with 10 months of treatment that was based on BPaLC with cycloserine. All components of the BPaL regimen achieved therapeutic concentrations in CSF for most TB strains. However, our data raise concerns that pretomanid and linezolid CNS concentrations may be subtherapeutic in TBM from strains with pretomanid or linezolid MIC >0.5 mg/L. Ongoing research is required into the effectiveness, optimal duration, and value of therapeutic drug monitoring of BPaL in CNS TB.
